# Proportion of medication error reporting and associated factors among nurses: a cross sectional study

**DOI:** 10.1186/s12912-018-0280-4

**Published:** 2018-03-12

**Authors:** Abebaw Jember, Mignote Hailu, Anteneh Messele, Tesfaye Demeke, Mohammed Hassen

**Affiliations:** 1 Department of Medical Nursing, School of Nursing, College of Medicine and Health Sciences, University of Gondar, Gondar, Ethiopia; 2 Unit of Community Health Nursing, School of Nursing, College of Medicine and Health Sciences, University of Gondar, Gondar, Ethiopia; 3 Department of Pediatric and Child Health Nursing, School of Nursing, College of Medicine and Health Sciences, University of Gondar, Gondar, Ethiopia

**Keywords:** Medication error, Medication error reporting, Federal Level Governmental Hospital, Nurse

## Abstract

**Background:**

A medication error (ME) is any preventable event that may cause or lead to inappropriate medication use or patient harm. Voluntary reporting has a principal role in appreciating the extent and impact of medication errors. Thus, exploration of the proportion of medication error reporting and associated factors among nurses is important to inform service providers and program implementers so as to improve the quality of the healthcare services.

**Methods:**

Institution based quantitative cross-sectional study was conducted among 397 nurses from March 6 to May 10, 2015. Stratified sampling followed by simple random sampling technique was used to select the study participants. The data were collected using structured self-administered questionnaire which was adopted from studies conducted in Australia and Jordan. A pilot study was carried out to validate the questionnaire before data collection for this study. Bivariate and multivariate logistic regression models were fitted to identify factors associated with the proportion of medication error reporting among nurses. An adjusted odds ratio with 95% confidence interval was computed to determine the level of significance.

**Result:**

The proportion of medication error reporting among nurses was found to be 57.4%. Regression analysis showed that sex, marital status, having made a medication error and medication error experience were significantly associated with medication error reporting.

**Conclusion:**

The proportion of medication error reporting among nurses in this study was found to be higher than other studies.

**Electronic supplementary material:**

The online version of this article (10.1186/s12912-018-0280-4) contains supplementary material, which is available to authorized users.

## Background

Medication therapy is the most common intervention prescribed for healthcare consumers [1] and safe medication administration represents one of the routine, highly complex and essential nursing care responsibilities [2]. Medication administration is amongst the potentially hazardous nursing tasks in hospitals because of liability to errors [3]. The reporting of error incidents and specific phases of healthcare delivery such as the safe use of medications can improve the safety of patients [4].

A single medication error (ME) may prolong hospital stay or even end up in death. This affects the quality and continuity of the healthcare services [5] by affecting patient safety which is an important indicator of healthcare quality and encompasses various nursing care procedures. Executing a ME has been seen to be psychologically devastating to the nurse and harmful to the patient [6].

Globally, MEs present a substantial contribution to ill health, and even death and are listed as one of the five medical error categories classified by the American Institute of Medicine [7]. Out of estimated patients’ deaths of 6000 to 20,000 each year from medical errors in Taiwan, 10% of medical lawsuits were because of MEs where the majority of the errors were grossly underreported [8]. A prospective cross-sectional study conducted in an intensive care unit of a specialized hospital in Ethiopia showed a ME of 51.8% [9].

Nevertheless how huge or insignificant the incidence, it is difficult to have a general concept of MEs in less developed and developing countries due to inefficient documentation and error-reporting systems and insufficient research in the area [7]. Analysis and appreciation of the root causes of MEs allows developing complex medication error prevention mechanisms [10] which improve patient safety.

## Literature review

A medication error is any preventable event that may cause or lead to inappropriate medication use or patient harm [11]. Medication error is a global issue where 5% of the MEs are deadly and almost 50% are preventable [12]. Medication error reporting is one of the major issues in today’s health care and prevention is linked to accurate reporting of errors [7]. Voluntary reporting is indispensable to appreciate the extent and impact of MEs [13]. Nurses’ interception of 86% of the MEs was presented in a descriptive cross-sectional study conducted in one large medical center hospital in southern Taiwan with sample size of 597 nurses using self-administered questionnaires and the significance of error reporting was given a weight as intercepting [8]. Moreover, consideration of nurses’ perceived barriers to medication error reporting (MER) is a crucial step to strengthen medication safety [8] and it was shown in a study that more than 90% of healthcare consumers believe that errors should be reported [3].

### Incidence and prevalence of medication errors

Medication errors which are made during prescription, dispensing and administration [14] are common and preventable causes of patient harm [15]. Precise figure of the incidence and prevalence of MEs is difficult to obtain because the rate varies from study to study [7]. Studies showed a range of rate of serious patient injuries due to medication errors as 1 to 2% [16], 9 to 13% [2], 29% [17], and as high of 51.8% [9], and estimated 30.5% deaths per year in a survey in the United States of America (USA) were attributable to MEs [15]. A study conducted in Southern Iran with the purpose of determining the frequency of MEs in an emergency department of a teaching hospital revealed that 96.5% of patients had experienced at least one medication error, making the rate of errors 3.5 per patient [18]. A descriptive survey of 300 nurses working in hospitals affiliated to Iran University of Medical Sciences using stratified multistage sampling disclosed a mean of 19.5 medication errors that the nurses acknowledged within 3-months period however the mean of error reporting was only 1.3 of error cases [19, 20].

### Factors related to medication error reporting

A focus group study on barriers to MER in Canada identified barriers as an individual, organizational and cultural [21]. According to a descriptive cross-sectional study with sample size of 799 nurses conducted in Jordan, the proportion of medication error reporting was relatively high among female nurses than male nurses [7]. Twenty-six percent of nurses in a study conducted in Israel indicated that all MEs in their wards were reported and 46% of the nurses showed self-reporting of MEs. The nurses emphasized on a personal barrier to non-reporting such as ME experiences and error reporting experiences [22].

A perception that incidence reports do not result in significant changes or benefits and errors that did not result in harm were among the factors that affected the attitude of nurses to report medication errors [23, 24]. Perceived barriers which affect attitude of nurses to report medication errors were fear of adverse consequences from reporting and being subjected to disciplinary actions, fear of being blamed, fear of reaction from the nurse manager, from peers and fear of loss of jobs [6–8, 19, 25]. Other barriers for not reporting MEs include nurses not being aware that an error had occurred, process of reporting (detailed paperwork, time constraints, not understanding incident reporting process), forgetting to make a report when the ward is busy, lack of time for reporting and lack of awareness of the importance of reporting [19, 23, 26].

Two-thirds (66.7%) of nurses involved in a study conducted in two state hospitals in Turkey who stated that they involved in medication errors in the preceding 6 months had not reported the errors. The reported reasons (social factors) for not reporting MEs included fear of consequences, fear of a culture of blame and the need to cover up for the colleague involved [27].

Modifiable barriers to MER for nurses reported in different studies as organizational factors were revealed as lack of feedback to the reporter, lack of a readily available MER system, lack of information on how to report a ME, no positive feedback for giving medication correctly, too much emphasis on ME as a quality indicator of nursing care and motivational factors (such as no encouragement by management, fear of loss of professional registration), lack of organizational leadership and support [3, 8, 19, 25, 28]. Similarly taking medical responsibility and fear of distrust from patients were barriers of medication error reporting [29].

**Fig. 1:**
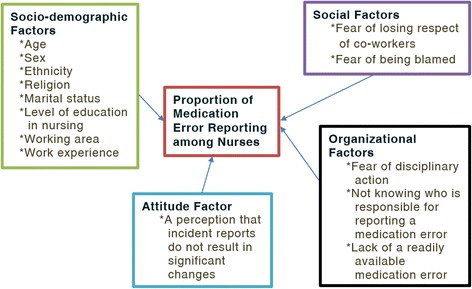
Conceptual framework of the factors related to the proportion of medication error reporting. [Source: Prepared by the investigator after thorough searching of literature]

Encouraging administrative attitudes and responses to MER were appreciated in a study to enhance nurses’ voluntary reporting [30]. It is indicated in the literature that strategies should be implemented to establish reporting mechanisms to reduce medication errors at national as well as international levels [31]. Establishing structured protocols on drug administration and adopting a non-punitive approach to reporting medication errors were shown to decrease medication errors and improve patient safety [32]. Proportion of medication error reporting by nurses might be affected by multiple factors such as socio-demographic, social, attitude of nurses, and organizational factors [7, 8, 12, 19] (Fig. 1 indicates the factors involved in medication error reporting).

Health care systems in most developing countries suffer from serious deficiencies in quality, equity, efficiency and financing [33]. The quality of care is evaluated in the light of the provider’s technical standards and clients’ expectations. Medication administration is among the routine and highly complex nursing care activities which plays a great role in patient care and outcome. Medication safety and medication errors are important concerns for health-care consumers, health-care professionals, researchers and policy makers worldwide.

Nurses are more prone to making medication errors because of the increasing demands and pressures placed on them. Critical incidents must be detected and reported and turned into positive situations, from which lessons are learned and used to design better patient care practices and systems. So far nurses are at the front line of defense to intercept and report medication errors, yet the errors are severely under-detected and under-reported in practice.

Medication errors affect the quality of health care delivery. Improving patient safety and learning from errors relies on voluntary error reporting which gives the complete picture of medication errors. Thus, exploration of the proportion of nurses reporting medication errors and associated factors is important to inform service providers, program implementers and policy makers to improve the quality of the healthcare service.

## Aim

The aim of this study was to assess the proportion of medication error reporting and to explore the relationships among the barriers; socio-demographic factors, organizational factors, social factors, and attitude of nurses.

Incident reporting represents more appropriate information about incidents and can detect preventable events if it is supported within the clinical settings. However, many of the incidents are not reported or are simply not recognized and yet it has been shown in the literature that nurses predominantly report incidents compared to other healthcare professionals [34]. Conversely, studies have demonstrated under-reporting of MEs among nurses [35].

## Methods

An institution based quantitative cross-sectional study was conducted at three Federal Ministry of Health level governmental hospitals located in the nation’s capital Addis Ababa from March 6 to May 10, 2015. There are four hospitals under the FMoH; Alert, St. Peter, St. Paul and Amanuel. Amanuel Mental Specialized Hospital is the only referral mental hospital in the country which delivers counseling and treatment for patients. St. Paul’s Millennium Medical College Hospital delivers medical services for an annual average of 200,000 patients. St. Peter’s TB Specialized Hospital has been giving services related to tuberculosis treatment. Alert Hospital was excluded because of protocol issues.

### Sample size determination

A single population proportion formula was used to calculate the sample size:

$$ n=\frac{{Z^2}_{\raisebox{1ex}{$\alpha $}\!\left/ \!\raisebox{-1ex}{$2$}\right.}p\left(1-p\right)}{d^2} $$

Where: n = minimum sample size required for the study.

z = score for 95% confidence interval (z = 1.96).

p = prevalence of medication error reporting (50%).

d = tolerable error (d = 5%).

Since the proportion of medication error reporting in Ethiopia is unknown, 50% prevalence of medication error reporting was taken:

$$ {n}_o=\frac{(1.96)^20.5\left(1-0.5\right)}{0.05^2} $$

$$ {n}_o=\frac{0.9604}{0.0025}=384.16\approx 385 $$

For possible non-response, the sample size was increased by 10%, thus comprising 38.416 respondents the final sample size became *423*.

### Sampling procedure

**Fig. 2:**
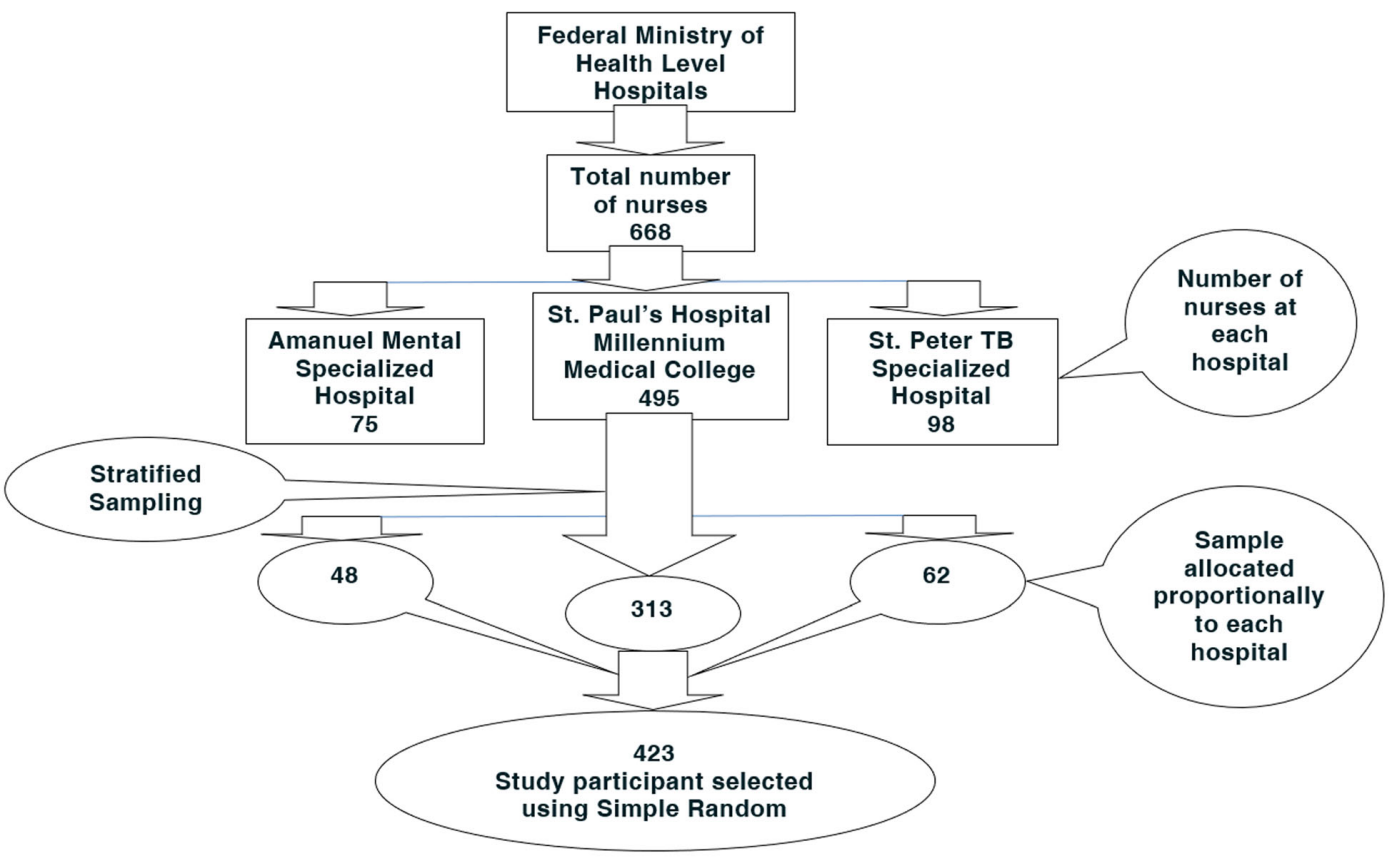
Schematic presentation of the sampling procedure to select the study participants

Stratified sampling technique was used to allocate the sample proportionally to each FMoH level governmental hospital and then the study participants who had work experience of six months and above were selected from the list of all nurses using simple random sampling technique; a lottery method (Fig. 2).

### Data collection tool and procedure

Data were collected by three nurses who were diploma holders using structured self-administered English-version questionnaire. The data collection tool was adopted from two studies conducted in Australia by Evans [3] and in Jordan by Mrayyan et al. [7] with the authors’ permissions and face and content validity were established by two expert nurse advisors. The tool was composed of five parts. The first part contained socio-demographic characteristics of nurses. The second part of the questionnaire was related to error incidence. The third part related to medication error reporting was used to estimate the proportion of medication error reporting. The fourth and fifth parts were used to collect data regarding the attitudes of nurses on medication error reporting and perceived organizational culture and reality of dealing with errors respectively (Additional file 1).

### Data quality control

Data quality was assured by conducting a pretest among 22 (5%) nurses at Zewditu hospital and appropriate modifications were made after analyzing the pre-test result before the actual data collection. The questionnaire was coded before data collection and cross-checked for consistency and completeness every day.

### Data management and analysis

The returned questionnaires were checked for completeness, cleaned and the data were entered into EPI Info-7 and then exported to SPSS (Statistical Package for the Social Sciences) version 20.0 for analysis. Frequencies and cross tabulations were used to summarize descriptive statistics and tables were used for data presentation. Binary logistic regression was used to identify factors associated with medication error reporting and then the variables were checked for significant association using *p*-value, odds ratio and 95% confidence interval.

## Result

### Socio-demographic characteristics

Out of 423 proposed study participants, 403 participated in the study giving a response rate of 95.27%. Six (1.41%) of the returned questionnaires were found to be incomplete and excluded and the rest 20 (4.72%) of nurses chose not to participate in the study. 397 (93.85%) of study participants’ responses were analyzed. The majority (53.7%) of respondents were male. The mean (+ standard deviation) age of the respondents was 28.37 (+ 5.55) years. 307 (77.3%) respondents were Orthodox Christian followed by Protestant 48 (12.1%), 208 (52.4%) of the respondents were Amhara in their ethnic background and 291 (73.3%) of the participants were single. On the other hand, 154 (38.8%) of the respondents worked in internal medicine wards and level of education of 237 (59.7%) of the participants was Bachelor of Science in Nursing. Moreover, 182 (45.8%) of the respondents served 1–3 years in the nursing profession (Table 1).

**Table 1:** Demographic characteristics of nurses at federal level teaching hospitals, Addis Ababa 2015 (*n* = 397)

Variables	n (%)
Sex	
Male	213 (53.7)
Female	184 (46.3)
Age	
19–29	312 (78.6)
30–39	52 (13.1)
40–49	21 (5.3)
50–59	12 (3.1)
Ethnicity	
Amhara	208 (52.4)
Oromia	93 (23.4)
SNNPR	60 (15.1)
Tigray	20 (5.0)
Others^a^	16 (4.0)
Religion	
Orthodox	307 (77.3)
Protestant	48 (12.1)
Muslim	36 (9.1)
Catholic	6 (1.5)
Marital Status	
Single^b^	291 (73.3)
Married	106 (26.7)
Level of education in nursing	
BSc	237 (59.7)
Diploma	160 (40.3)
Working area	
Internal medicine ward	154 (38.8)
Surgical ward	73 (18.4)
Emergency room	63 (15.9)
Psychiatry	47 (11.8)
Intensive care unit	31 (7.8)
Pediatric ward	29 (7.3)
Service year in the nursing profession	
< 1	54 (13.6)
1–3	182 (45.8)
4–5	67 (16.9)
6–10	62 (15.6)
> 10	32 (8.1)

^a^Others: Benishangul-Gumuz, Harari, Gambella

^b^Single includes divorced and widowed

### Proportion of medication error reporting

The proportion of medication error reporting among nurses in this study was found to be 57.4% (*n* = 288). Out of the total participants (*n* = 397), relatively high (74.5%) proportion of medication error reporting was found among female nurses as compared to male nurses (42.7%). A high proportion (70.8%) of medication error reporting was disclosed by married participants than participants who are single (52.6%). On the other hand, 277 (69.8%) of the participants perceived that medication errors should be reported as they occur. Moreover, (70.7%) of the nurses who did not make errors themselves reported medication errors.

### Factors associated with medication error reporting

In bivariate logistic regression analysis sex, marital status, having made a medication error, medication error experience, and working area were associated with medication error reporting. However, in multivariate analysis sex, marital status, having made a medication error and medication error experience were associated with medication error reporting (Table 2).

**Table 2:** Bivariate and multivariate logistic regression analysis of factors associated with proportion of medication error reporting among nurses at selected Federal Ministry of Health level hospitals, Addis Ababa 2015 (*n* = 397)

Variables	Medication error reporting practice		Odds Ratio (95% CI)	
	Yes	No	Crude	Adjusted
Sex				
Male	91	122	1.00	1.00
Female	137	47	0.256 (0.167–0.393)	0.273 (0.165–0.450)^*^
Marital status				
Single	153	138	1.00	1.00
Married	75	31	0.458 (0.284–0.739)	0.454 (0.251–0.821)^*^
Medication error experience				
Yes	94	104	1.00	1.00
No	134	65	0.438 (0.292–0.659)	0.445 (0.274–0.722)^*^
I made medication errors				
Yes	170	145	1.00	1.00
No	58	24	0.485 (0.287–0.820)	0.426 (0.230–0.789)^*^
Should errors be reported				
Yes	132	145	1.00	1.00
No	96	24	0.228 (0.137–0.377)	0.151 (0.082–0.277)^*^
Error reporting leads to beneficial and constructive activity				
Yes	197	159	1.00	1.00
No	31	10	0.400 (0.190–0.840)	0.881 (0.334–2.322)
Working area: Intensive care unit				
Yes	39	24	1.00	1.00
No	8	23	4.672 (1.804–12.101)	4.471 (0.502–13.307)
Religion: Protestant				
Yes	165	142	1.00	1.00
No	34	14	0.478 (0.247–0.927)	0.672 (0.296–1.525)

^*^Statistically significant at *P* < 0.05

The proportion of medication error reporting was high among female nurses as compared to male nurses. Female nurses were 72.7% times more likely to report medication errors than male nurses (AOR = 0.273; 95% CI = 0.165–0.450). Similarly, marital status was an important predictor of medication error reporting. Nurses who are married were 54.6% times less likely to report medication errors as compared to those who are single (AOR = 0.454; 95% CI = 0.251–0.821).

Having a medication error experience was found to be another important determinant of medication error reporting. Nurses who had no medication error experience were 55.5% times more likely to report medication errors than those who had medication error experiences (AOR = 0.445; 95% CI = 0.274–0.722). The odds of medication error reporting among nurses who have not made errors themselves was 57.4% times higher as compared to those nurses who had previously made medication errors (AOR = 0.426; 95% CI = 0.230–0.789).

## Discussion

The proportion of medication error reporting among nurses was found to be 57.4%. This finding is slightly higher as compared to studies in Jordan 42.1% [7], Australia 41.9% [3] and California, USA 28.9% [6]. The possible differences could be related to the differences in organizational medication error reporting systems and differences in the time frame that the studies are bounded.

In this study medication error experience of nurses was significantly associated with medication error reporting. Nurses who had previous experiences of medication errors and their reporting processes that errors made by others were 55.5% less likely to report error incidents than those who did not have error experiences and those nurses who had previously made medication errors were 57.4% less likely to report error incidents than those who did not make medication errors. This result is consistent with the result from Israel [22] and South Korea [26] that ME and having made errors hindered nurses from reporting MEs. However, this result was inconsistent with other results from Taiwan [8], Southern Taiwan [29] and Saudi Arabia [25]. The difference may be related to the severity of medication errors since minor errors are less likely to be reported, even though it was difficult to determine the effect of medication errors experienced regarding severity.

Most of the participants (69.8%) perceived that errors should be reported as they occur for the safety of patients and this was consistent with the study from the United Kingdom [23] however lower than the study from Taiwan (87.7%) [8]. The possible difference may be due to the lack of a readily available practice system of medication error reporting.

In this study, the proportion of female nurses who reported medication errors was higher than the male nurses and was statistically significant. The result was similar with that of the study from Jordan [7]. In contrast to other studies, marital status in this study was statistically significant with error reporting (AOR = 0.454; 95% CI = 0.251–0.821) showing that nurses who are married were 54.6% less likely to report medication errors. This might be related to barriers like fear of disciplinary actions since it was put in second place as perceived barrier to reporting by nurses though not statistically significant.

## Conclusion

The proportion of medication error reporting among nurses in this study was found to be high. Medication error experience, having made a medication error, sex of participants and marital status were significantly associated.

### Limitation

Causes of medication errors were not studied in this research. Knowing the causes of medication errors gives a complete picture of medication errors. Therefore, it would be better if the study was triangulated. Response bias and recall bias were not reduced.

### Recommendation

#### To Federal Ministry of Health

It is recommended that the Federal Ministry of Health identify and address gaps in medication error identification and reporting regarding the establishment of the reporting system to improve patient safety.

#### To the respected hospitals

The respective hospitals efforts will be significant in identifying and addressing gaps in medication error reporting among nurses and creating a conducive environment for the establishment and maintenance of efficient reporting system. A condition in which reported errors are changed into opportunities to learn from them.

#### To the respected nurses

Nurses’ contribution to the documentation of errors and reporting is an important input for the smooth provision of quality care and improved patient outcome.

#### To researchers

Further investigation, qualitative in nature, should be made in order to have a complete picture of medication errors in Ethiopia.

## Additional file

Additional file 1: Questionnaire Medication Error Reporting. Questionnaire. Questionnaire prepared to collect data for the assessment of the intent of nurses to report medication errors and associated factors at federal level governmental teaching hospitals in Addis Ababa, Ethiopia 2015. (DOCX 26 kb)

## Abbreviations

FMoH: Federal Ministry of Health
ME: Medication error
MER: Medication error reporting
MoH’: Ministry of Health
USA: United States of America
