# Supplementary material for: Proportion of medication error reporting and associated factors among nurses: a cross sectional study
Source: BMC Nurs. 2018 Mar 12;17:9. doi: 10.1186/s12912-018-0280-4 (PMC5848571; doi:10.1186/s12912-018-0280-4)
Supplement: Supplementary file 1 — Questionnaire Medication Error Reporting. Questionnaire. Questionnaire prepared to collect data for the assessment of the intent of nurses to report medication errors and associated factors at federal level governmental teaching hospitals in Addis Ababa, Ethiopia 2015. (DOCX 26 kb) [file 12912_2018_280_MOESM1_ESM.docx]

**Questionnaire**

**Questionnaire prepared to collect data for the assessment of the intent of nurses to report medication errors and associated factors at federal level governmental teaching hospitals in Addis Ababa, Ethiopia 2015**

This questionnaire is used to collect data from nurses who work in this hospital to assess the intent of medication error reporting and associated factors. Participants are selected randomly. Confidentiality of the participants will be maintained by making the questionnaire anonymous and keeping the data generated only accessible by the investigator.

There may not be immediate, direct and financial benefits for the participants, but nurses might benefit from the nursing knowledge gained through the process. There are no potential harms that impact on employment or social status for participating in this study. The data generated will be used only for the intended research and the results of the study will be published in a reputable journal with no identifiable information.

Ethical clearance and approval was obtained from Ethical Review Committee of School of Nursing, College of Medicine and Health Sciences, University of Gondar and permission was obtained from the Ethical Review Committee of the hospital.

Your role in the success of the research is immense and I appreciate for your accurate and honest responses and the contribution you made to this study.

**Consent**

I have been explained about the risk and benefits of the research and the roles I will have in the research. I agree to participate in the study.

1. Yes B. No

**Thank you for voluntarily participate in the study!**

**Instructions**

There are series of questions to be answered. You are expected to respond to all. It will take 15 minutes to complete this questionnaire. There is no need of writing your name. You can refuse to respond to any of the questions and interrupt at any point. You can ask questions at any point if you have any.

Identification number: ________

**Principal Investigator:** Abebaw Jember

Telephone: +251-934-538090

Email: [abebaw5360@gmail.com](mailto:abebaw5360@gmail.com)

| 1. **Socio-demographic information** | | | |
| --- | --- | --- | --- |
| **Ser. No** | **Query categories** | **Response categories** | **Remark** |
| 101 | Sex | Male1  Female2 |  |
| 102 | Age in years | ______ |  |
| 103 | Ethnicity | Afar1  Amhara2  Benishangul-Gumuz3  Gambela4  Harari5  Oromia6  Somali7  SNNPR8  Tigray9 |  |
| 104 | Religion | Orthodox1  Muslim2  Catholic3  Protestant....4  Others5 |  |
| 105 | Marital status | Married1  Single2  Divorced3  Widowed4  Others5 |  |
| 106 | Level of education in nursing | Diploma1  BSc2  MSc3  PhD4 |  |
| 107 | Working area | Internal medicine ward1  Surgical ward2  Paediatric ward3  Intensive care unit4  Emergency room5  Psychiatry6 |  |
| 108 | How long have you been in the nursing profession? | _______ Years / Months |  |

| 1. **Questions related to Errors incidence** | | | |
| --- | --- | --- | --- |
| 201 | Have you ever experienced any medication administration error? | Yes1  No2  If your answer for this question is ‘No’ jump to question 301 |  |
| 202 | I had encountered cases of incorrect medication treatment that I gave to my patient. | Yes1  No2 |  |
| 203 | I discovered medication error cases that others committed. | Yes1  No2 |  |
| 204 | I intercepted cases where patients were about to receive incorrect medication. | Yes1  No2 |  |
| 205 | Which kind of medication error did you encounter? | Omission1  Wrong dosage2  Wrong time3  Wrong frequency4  Prescribing5  Wrong patient6  Wrong site7  Dispensing8 |  |
| 1. **Questions related to error self-reporting:** | | | |
| 301 | Should medication errors be reported? | Yes1  No2 |  |
| 302 | Have you ever reported a medication error? | Yes1  No2 |  |
| 303 | To whom do you prefer reporting medication errors? | Team leader nurse1  Nursing director2  Intern doctor3  Medical director4  Concerned specialist5 |  |
| 304 | Which error do you prefer to report? | One should always report all medication errors he/she had done.1  One should always report all medication errors made by others2  One should always report all errors he/she discovers3 |  |
| 305 | Is there a reporting protocol in your hospital? | Yes1  No2 |  |

| 1. **Questions related to attitudes on error reporting:** | | | | |
| --- | --- | --- | --- | --- |
| 401 | What causes do you think hinder nurses from reporting medication errors? | Lack of a readily available medication error reporting system1  Lack of knowledge of which medication errors should be reported2  Lack of knowledge of the usefulness of reporting medication errors3  Fear of disciplinary action4  Fear of being blamed5  Not knowing who is responsible for reporting a medication error6  Belief that it is unnecessary to report medication errors not associated with patient harm7  Fear of losing respect of co-workers8 | |  |
| 1. **Perceived organizational culture and reality of dealing with errors:** | | | | |
| 501 | Medication error discovering and reporting leads to a beneficial and constructive activity. | | Strongly agree1  Agree2  Neither agree nor disagree3  Disagree4  Strongly disagree5 |  |
| 502 | Discovering a medication error has a negative impact on the nurse's possible development and career in organizational hierarchy. | | Strongly agree1  Agree2  Neither agree nor disagree3  Disagree4  Strongly disagree5 |  |

***Thank You Again!***
